# RAMPred: identifying the N^1^-methyladenosine sites in eukaryotic transcriptomes

**DOI:** 10.1038/srep31080

**Published:** 2016-08-11

**Authors:** Wei Chen, Pengmian Feng, Hua Tang, Hui Ding, Hao Lin

**Affiliations:** 1 Department of Physics, School of Sciences, and Center for Genomics and Computational Biology, North China University of Science and Technology, Tangshan, Tangshan 063000, China; 2 School of Public Health, North China University of Science and Technology, Tangshan, 063000, China; 3 Department of Pathophysiology, Southwest Medical University, Luzhou 646000, China; 4 Key Laboratory for Neuro-Information of Ministry of Education, Center of Bioinformatics and Center for Information in Biomedicine, School of Life Science and Technology, University of Electronic Science and Technology of China, Chengdu 610054, China

## Abstract

N^1^-methyladenosine (m^1^A) is a prominent RNA modification involved in many biological processes. Accurate identification of m^1^A site is invaluable for better understanding the biological functions of m^1^A. However, limitations in experimental methods preclude the progress towards the identification of m^1^A site. As an excellent complement of experimental methods, a support vector machine based-method called RAMPred is proposed to identify m^1^A sites in *H. sapiens*, *M. musculus* and *S. cerevisiae* genomes for the first time. In this method, RNA sequences are encoded by using nucleotide chemical property and nucleotide compositions. RAMPred achieves promising performances in jackknife tests, cross cell line tests and cross species tests, indicating that RAMPred holds very high potential to become a useful tool for identifying m^1^A sites. For the convenience of experimental scientists, a web-server based on the proposed model was constructed and could be freely accessible at http://lin.uestc.edu.cn/server/RAMPred.

The N^1^-methyladenosine (m^1^A) is a prominent post-transcriptional modification found in RNA, which is catalyzed by methyltransferase^1^. Besides adding a methyl group to the nitrogen at the 1st position of the adenosine base, m^1^A also endows the modified adenosine with a positive charge^2^, as shown in Fig. 1. It has been found that m^1^A has major influences on the structure and function of tRNA and rRNA^3^,^4^,^5^. For example, m^1^A in tRNA can respond to environmental stress^6^,^7^, and m^1^A in rRNA can affect ribosome biogenesis^8^ and mediate antibiotic resistance in bacteria^9^. Although the functions of m^1^A in tRNA and rRNA were well studied, similar researches in mRNA were precluded due to the lack of effective methods for detecting m^1^A in mRNA^2^,^10^. Therefore, the knowledge about the positions of m^1^A site is important for understanding mechanisms and functions of this post-transcriptional modification.

With the development of high-throughput experimental techniques, such as MeRIP-seq^2^ and m^1^A-ID-seq^10^, high-resolution m^1^A maps are available for *H. sapiens*, *M. musculus* and *S. cerevisiae* transcriptomes^2^. These experimental results revealed that m^1^A sites are enriched in 5′-untranslated region and coding sequence of mRNA transcripts^2^,^10^, and also demonstrated that m^1^A is dynamic in response to physiological conditions and correlates positively with protein production^2^.

Experimental methods did play a role in promoting the research progress on identifying m^1^A sites. However, their resolutions are not fully satisfactory, i.e. they cannot pinpoint which adenosine residue is actually modified^10^. Therefore, it is necessary to develop new methods for studying the distribution of m^1^A site. As excellent complements to experimental techniques, computational methods will speed up genome-wide m^1^A detection.

The high-resolution experimental data provided unprecedented opportunities and made it feasible to develop computational methods for accurately predicting m^1^A sites. However, to the best of our knowledge, there is no computational tool available for the identification of m^1^A. Hence, in the present study, we propose a support vector machine based-method to identify the m^1^A sites in the *H. sapiens*, *M. musculus* and *S. cerevisiae* genomes. By using the nucleotide chemical property and nucleotide composition, the sequence-order effects and nucleotide chemical properties are integrated together in the proposed model. It is encouraging that the proposed methods obtained promising performances in jackknife tests, cross cell line tests and cross species tests. For the convenience of scientific community, a web-server for the proposed model is provided at http://lin.uestc.edu.cn/server/RAMPred.

## Result and Discussion

### m^1^A sites identification

In statistical prediction, three cross-validation methods, i.e., independent dataset test, sub-sampling (or n-fold cross-validation) test, and jackknife test, are often used to evaluate the anticipated success rate of a predictor. Among the three methods, the jackknife test is deemed the least arbitrary and most objective^11^. Therefore, the jackknife test has been increasingly adopted by researchers to examine the quality of various computational models^12^,^13^,^14^,^15^,^16^. Thus, the jackknife test was used to examine the performance of the proposed model. In the jackknife test, each sample in the training dataset is in turn singled out as an independent test sample and all the properties are calculated without including the one being identified.

**Figure 2:**
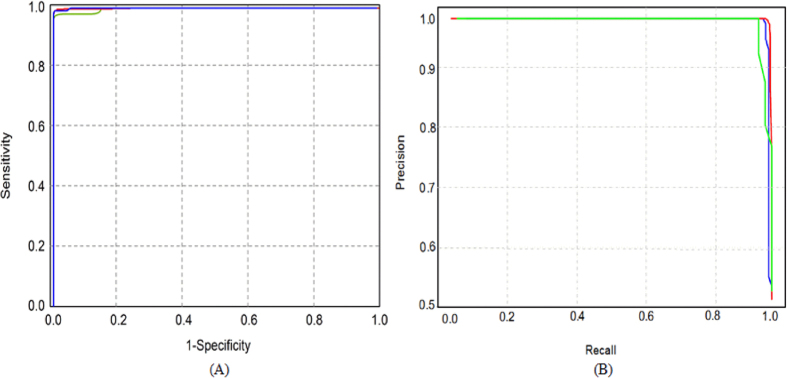
A graphical illustration to show the performance of RAMPred for identifying m^1^A sites in *H. sapiens* (red line), *M. musculus* (blue line) and *S. cerevisiae* (green line) genomes. The performances are illustrated by means of the ROC curves (**A**) and precision-recall curves (**B**).

By encoding RNA sequence using nucleotide chemical property and nucleotide composition, each 41-bp long sequence in the dataset was transferred to a 164 (4 × 41)-dimensional vector (see Materials and Methods) and was used as the input of SVM. The model thus obtained is called RAMPred (RNA N^1^-adenosine methylation predictor). The jackknife test results of RAMPred for identifying m^1^A sites in *H. sapiens*, *M. musculus* and *S. cerevisiae* genomes were enumerated in the first four columns of Table 1. In addition, in order to objectively evaluate the performance of RAMPred in identifying m^1^A sites, the receiver operating characteristic curves and precision recall curves for *H. sapiens*, *M. musculus* and *S. cerevisiae* were also plotted and were shown in Fig. 2. The AUROC and AUPRC values examining the performance of RAMPred were calculated and provided in the last two columns of Table 1. As we can see from Table 1 and Fig. 2, the prediction accuracies of RAMPred were considerably high for identifying m^1^A sites in all the three species.

**Figure 3:**
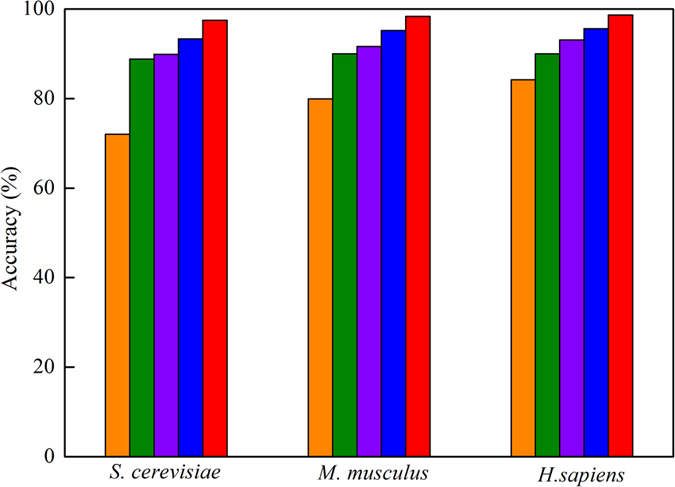
The predictive accuracies obtained from the jackknife test for identifying m^1^A sites in *H. sapiens*, *M. musculus* and *S. cerevisiae* genome by using different kinds of parameters. Orange, green, purple, blue and red histograms stand for the accuracies obtained by the model trained by using nucleotide composition, chemical functionality, hydrogen bond, ring structure and the combination of all the four kinds of features, respectively.

The chemical properties or nucleotide composition may have different roles in the prediction of m^1^A site. In order to investigate the contribution of each feature for m^1^A site identification, we built a series of models and validated them on the benchmark dataset. Their predictive accuracies obtained from jackknife test for identifying m^1^A sites in *H. sapiens*, *M. musculus* and *S. cerevisiae* genomes were shown in Fig. 3. It was observed that, among the four kind of features (namely ring structure, hydrogen bond, chemical functionality and nucleotide composition), the model based on the ring structure yields the highest accuracy. However, it’s lower than that obtained by using their combinations (Fig. 3). These results indicate that ring structure has the largest contribution for m^1^A site identification in the current method, and the other three features (hydrogen bond, chemical functionality and nucleotide composition) play complementary roles for the prediction.

In addition, to ensure that the predictive accuracy of RAMPred is not sensitive to the selection of negative data, we repeated the random sampling procedure ten times. In each time, a prediction model was built based on the positive dataset and the generated negative dataset. For saving computational time, the four metrics as defined in Eq. 4 for the other nine models in identifying m^1^A sites via the 10-fold cross validation test were reported in Supplementary Tables S1–S3 for *H. sapiens*, *M. musculus* and *S. cerevisiae*, respectively. We found that the predictive accuracy is not affected by the selection of negative data, demonstrating the reliability and robustness of the model proposed in this study.

### Comparison with Other classifiers

To the best of our knowledge, there is no published computational method for identifying m^1^A sites. We could not provide the comparison analysis with existing results to confirm that RAMPred is superior to other methods. However, to further testify its superiority, the predictive results of RAMPred were compared with that of other commonly used classifiers, i.e., J48 Tree, Random Forest, Naïve Bayes and BayesNet as implemented in WEKA^17^. For saving computational time, the 10-fold cross validation test results of different classifiers for identifying m^1^A in the benchmark dataset were reported in Supplementary Table S4. It is shown that the four metrics as defined in Eq. 4 for RAMPred are all higher than those of J48 Tree, Random Forest, Naïve Bayes and BayesNet.

Recently, Chen *et al.* proposed the iRNA-Methyl tool to identify post-transcriptional RNA modifications^18^. In iRNA-Methyl, RNA sequence was formulated with the “pseudo dinucleotide composition” (PseDNC)^19^,^20^,^21^ into which three RNA physical-chemical properties (i.e. enthalpy, entropy and free energy) were incorporated^18^. To demonstrate the effectiveness of nucleotide chemical properties and nucleotide composition for m^1^A site identification, the PseDNC-based SVM model was also developed. The 10-fold cross validation test results of the PseDNC-based SVM model in identifying m^1^A site by using the same benchmark dataset are given in Supplementary Table S5, from which we can see that the performance of RAMPred is superior to the PseDNC-based SVM model for identifying m^1^A site. All these results indicate that RAMPred can be effectively used to identify m^1^A sites.

### Cross cell line and cross species validation

**Figure 4:**
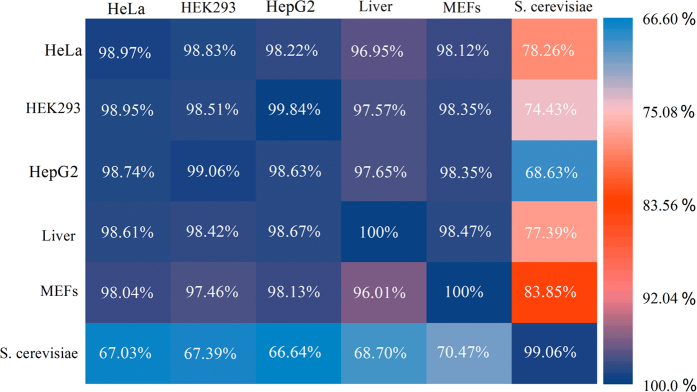
The heat map showing the cross cell line and cross species prediction accuracies. The accuracies were evaluated through the independent tests. More specifically, once a cell line or species model was established on its own training dataset, it was tested on the data from the same cell line (or species) as well as those from the other cell lines (or species). In cross species analysis, we considered three kinds of species (i.e., *H. sapiens*, *M. musculus* and *S. cerevisiae*). In cross cell line analysis, we considered three kinds of human cell lines (i.e., HeLa, HEK293 and HepG2) and two kinds of mouse cell lines (i.e., Liver and MEFs).

m^1^A is a dynamic modification in response to certain stress conditions and its level varies among different tissues^2^. Since the training dataset of RAMPred were collected from different species and cell lines (see Materials and Methods), it is interesting to see to what extent a model trained by using the data from one tissue or specie recognizes the m^1^A sites from other tissues or species. To demonstrate this point, we trained cell line-specific and species-specific models based on the m^1^A site data from different cell lines and species, and then validated them on the independent datasets from other cell lines or species. The cross cell line and cross species independent test results are given in Fig. 4.

It was found that the mammalian models trained using data from *H. sapiens* and *M. musculus* genomes can accurately identify each other’s m^1^A sites and the performances are pretty good. Although the performances of the mammalian models for identifying m^1^A sites in yeast genome are acceptable, they are lower than that obtained by the model trained using data from yeast own data. This result indicates that the construction of species-specific predictor for identifying m^1^A sites is necessary for yeast. It was also found that the cross cell line prediction performances are satisfactory and are equivalent to the intra-cell line performances in the three human cell lines (i.e., HeLa, HEK293 and HepG2) and two mouse cell lines (i.e., Liver and MEFs), indicating that there is no need to construct cell line-specific models to identify m^1^A sites in mammalian genomes.

### Web-Server and User Guide

To enable applications of the proposed method and for the conveniences of community, a freely accessible online web-server called RAMPred was established. To maximize the user’s convenience, a step-by-step guide of the web-server on how to use RAMPred is given as following.

**Figure 5:**
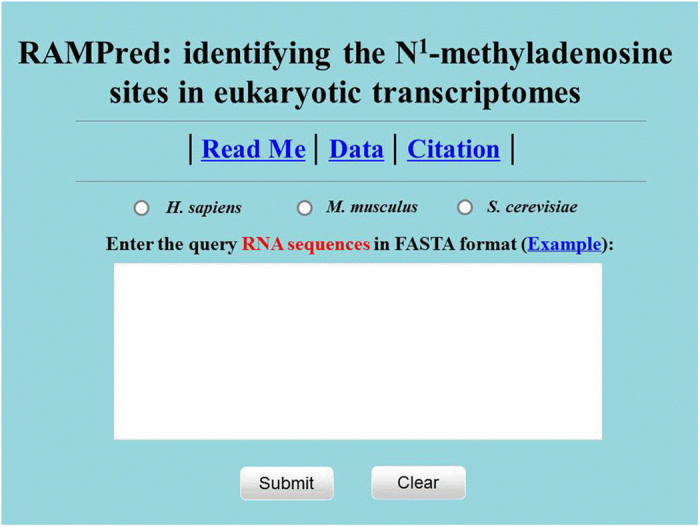
A semi-screenshot for the top-page of the RAMPred web-server at http://lin.uestc.edu.cn/server/RAMPred.

Firstly, browse the web server at http://lin.uestc.edu.cn/server/RAMPred and you will see the top page of RAMPred on your computer screen, as shown Fig. 5. Click on the Read Me button to see a brief introduction about the predictor and the caveat when using it. Click on the Data button to download the benchmark datasets used to train RAMPred. Click on the Citation button to find the relevant papers that document the detailed development and algorithm of RAMPred.

Secondly, select the organism or species by checking on the corresponding open circle. To get the anticipated prediction accuracy, the species button must be consistent with the source of query sequences: if the query sequences are from *H. sapiens*, check on the ‘*H. sapiens*’ button; if from *M. musculus*, check on the ‘*M. musculus*’ button; if from *S. cerevisiae*, check on the ‘*S. cerevisiae*’ button. Either type or copy/paste the query RNA sequences into the input box at the center of Fig. 5. The input sequence should be in FASTA format. For the examples of RNA sequences in FASTA format, click the Example button right above the input box. The predicted results will be shown on the computer screen by clicking on the Submit button.

## Conclusions

By using nucleotide chemical property and nucleotide composition, for the first time, we developed a support vector machine-based model to identify m^1^A sites in *H. sapiens*, *M. musculus* and *S. cerevisiae* genomes. The jackknife test results on the rigorous benchmark datasets demonstrate that the proposed method RAMPred is very promising for identifying m^1^A sites in the three eukaryotic genomes.

To identify the key features for m^1^A site identification, we compared the predictive results obtained by using different kind of parameters and found that the ring structure has the largest contribution for m^1^A site identification. This result holds for all the three genomes and is consistent with the following fact. N^1^-methylation on RNA adenosine occurs at the Watson-Crick interface and is catalyzed by methyl-transferases that need to recognize and bind with specific genomic regions^22^. Therefore, nucleotide ring structure could facilitate the π-cation/π-π/van der Waals contact between methyl-transferases and RNA sequence.

In order to rigorously evaluate its performance, we also tested the proposed method by performing cross cell line and cross species validations. It is encouraging to see that the cross cell line performances are quite good, indicating that our method is stable for identifying m^1^A site in mammalian genomes. We also noticed that the performances of mammalian based models for identifying yeast m^1^A sites are lower than that of the yeast specific one and vice versa.

As an epigenetic modification, RNA methylation is a complicate progress. Besides sequence context and nucleotide chemical properties, other factors may be also helpful for m^1^A site identification. For example, it has been demonstrated that m^1^A correlates with elevated translation, and enriched in 5′-untranslated region and coding sequence, and also overrepresented in the start codon upstream of the first splice site^2^,^10^. In addition, high-resolution experimental data with quantitative information about m^1^A modification are also highly desirable, which would aid the representation of the sequence context surrounding the m^1^A sites. For better understanding of the biological function of N^1^-methylation on RNA adenosine, we will combine all these factors and develop new models to improve the predictor’s performance for m^1^A sites identification in the future work.

## Materials and Methods

### Datasets

Based on MeRIP-seq technique, Dominissini and his colleagues obtained the m^1^A peaks in *H. sapiens*, *M. musculus* and *S. cerevisiae* genomes^2^. By mapping these peaks to *H. sapiens* (hg.19), *M. musculus* (mm10) and *S. cerevisiae* genome, respectively, we obtained m^1^A site containing sequences for these three genomes. It was observed via preliminary trials that when the length of the sequences in the benchmark dataset is 41 bp with the m^1^A in the center, the corresponding predictive results were most promising. Accordingly, we focus on RNA sequence with 41 nucleotides in the current study.

To overcome redundancy and reduce the homology bias, sequences with more than 80% sequence similarity were removed by using the CD-HIT program^23^. After such a screening procedure, we obtained 6,366, 1,064 and 483 m^1^A site containing sequences and deemed them as the positive samples of *H. sapiens*, *M. musculus* and *S. cerevisiae*, respectively. If the sequence identity is set to a lower percentage, such as 40%, the result will be more objective and reliable. However, in this study we did not use such a stringent criterion because the currently available data do not allow this. Otherwise, the number of samples will be too few to have statistical significance.

The negative samples in each species were obtained by choosing the 41-nt long sequences satisfying the rule that the adenosine in the center was not detected by the MeRIP-seq technique. By doing so, we could obtain a great number of negative samples in each species. Therefore, the number of negative samples will be dramatically larger than those of positive samples. In machine-learning problems, imbalanced datasets can significantly affect the performance evaluation of learning methods. To balance out the numbers between positive and negative samples in model training, we randomly picked out 6,366, 1,064 and 483 sequences to form the negative samples for *H. sapiens*, *M. musculus* and *S. cerevisiae*, respectively. To demonstrate the robustness of the proposed model, we repeated the random sampling procedure ten times and obtained ten random samples of negative datasets for downstream training and prediction for each species.

**Figure 6:**
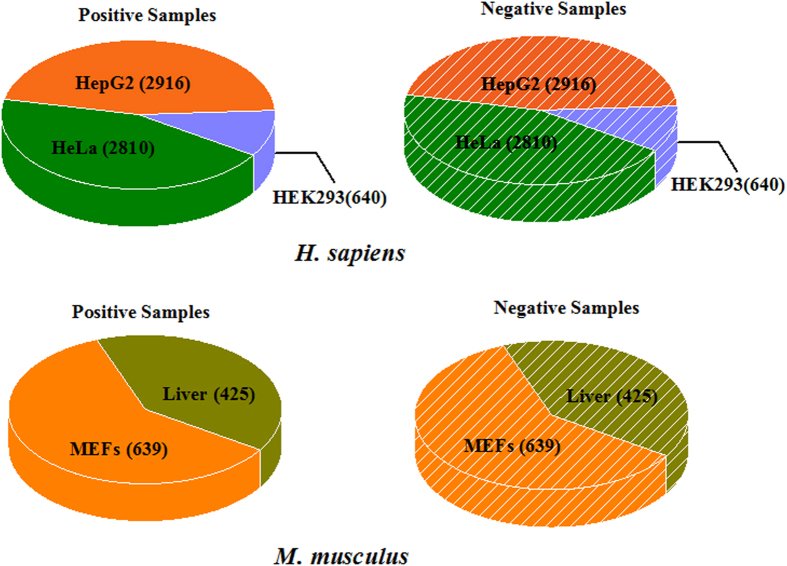
A graph to show the number of positive and negative samples in *H. sapiens* (top panel) and *M. musculus* (down panel) different cell lines. The left panel is the number of positive samples in each cell line, which is enumerated in brackets of the pie chart. The right panel is the number of negative samples in each cell line, which is enumerated in brackets of the pie chart.

According to Dominissini and his colleagues’ work^2^, the m^1^A site containing sequences in *H. sapiens* were from three cell lines, namely, HeLa (cervical adenocarcinoma), HepG2 (hepatocellular carcinoma) and HEK293 (embryonic kidney) cell lines, and those sequences in *M. musculus* were from two cell lines, namely, primary mouse embryonic fibroblasts (MEFs) and liver cell lines. To further validate the performance of the proposed method, we also built cell line specific datasets for *H. sapiens* and *M. musculus*, respectively. The numbers of positive and negative samples of the cell line specific datasets were shown in Fig. 6. All the data are available at http://lin.uestc.edu.cn/server/RAM/data.

### Representation of RNA sequences

Stimulated by its success in identifying post-transcriptional RNA modifications^24^,^25^, nucleotide chemical property and nucleotide composition were used to represent RNA sequences for identifying m^1^A sites in the present work. Below is the brief elaboration on how to encode RNA sequences using nucleotide chemical property and nucleotide composition.

RNA is transcribed with four nucleotides, namely, adenine (A), guanine (G), cytosine (C) and uracil (U). These four bases have different chemical properties. In terms of ring structures, adenine and guanine are purines that have two rings, while cytosine and uracil are pyrimidines that have one ring. When forming secondary structures, guanine and cytosine form strong hydrogen bonds, whereas adenine and uracil form weak hydrogen bonds. In terms of chemical functionality, adenine and cytosine can be classified into the amino group, while guanine and uracil into the keto group.

In order to include these chemical properties in RNA encoding, three coordinates (*x*, *y*, *z*) were used to represent the chemical properties of the four nucleotides and were assigned 1 or 0 values^24^,^26^. The *x* coordinate stands for the ring structure, *y* for the hydrogen bond, and *z* for the chemical functionality. Hence, each nucleotide in RNA sequence can be encoded by (*x*_*i*_, *y*_*i*_, *z*_*i*_), where^24^,^25^


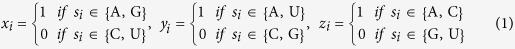


Thus, nucleotides A, C, G and U can be represented by the coordinates (1, 1, 1), (0, 0, 1), (1, 0, 0) and (0, 1, 0), respectively.

For the purpose of including nucleotide composition surrounding the m^1^A sites as well^2^, the density *d*_*i*_ of any nucleotide *n*_*j*_ at position *i* in a RNA sequence was defined by the following formula.


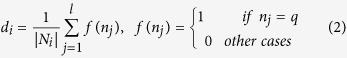


where *l* is the sequence length, |N_i_| is the length of the *i*-th prefix string {*n*_1_, *n*_2_, …, *n*_*i*_} in the sequence, *q* ∈ {A, C, G, U}.

**Figure 7:**
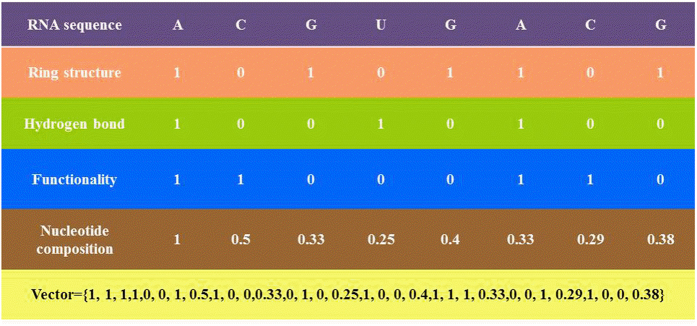
Scheme of encoding RNA sequence using nucleotide chemical properties and nucleotide composition.

Therefore, by integrating nucleotide chemical properties and nucleotide composition, the sequence with a length of *l* will be encoded by a (4 × *l*)-dimensional vector. An example of encoding RNA sequence using nucleotide chemical properties and nucleotide composition is shown in Fig. 7.

### Support Vector Machine

Support vector machine (SVM) is a powerful and popular method for pattern recognition and is widely used in the realm of bioinformatics^18^,^27^,^28^,^29^. The basic idea of SVM is to transform the input data into a high dimensional feature space and then determine the optimal separating hyperplane. In the current study, the LibSVM package 3.18 ( http://www.csie.ntu.edu.tw/~cjlin/libsvm/) was used to implement SVM. Due to its effectiveness and speed in training process, the radial basis kernel function (RBF) was used to obtain the classification hyperplane in the current study. In the SVM operation engine, the grid search method was applied to optimize the regularization parameter *C* and kernel parameter *γ* using a grid search approach as defined by


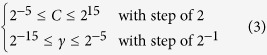


### Performance evaluation

The performance of the proposed method was evaluated by using the following four metrics, namely sensitivity (*Sn*), specificity (*Sp*), Accuracy (*Acc*) and the Mathew’s correlation coefficient (*MCC*), which are expressed as


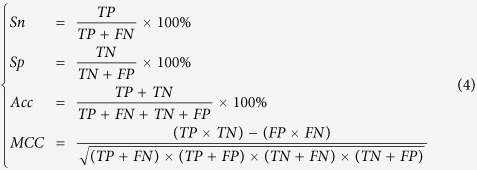


where *TP*, *TN*, *FP*, and *FN* represent true positive, true negative, false positive, and false negative, respectively.

The ROC (receiver operating characteristic) curve^30^ was also used to evaluate the performance of the current method, which plots the true positive rate (sensitivity) against the false positive rate (1-specificity). A best possible prediction method would yield a point with the coordinate (0, 1) representing 100% sensitivity and 0 false positive rate or 100% specificity. Therefore, the (0, 1) point is also called a perfect classification. A completely random guess would give a point along a diagonal from the point (0, 0) to (1, 1). The area under the ROC curve, also called AUROC, is often used to indicate the performance quality of a binary classifier: the value 0.5 of AUROC is equivalent to random prediction while 1 of AUROC represents a perfect one. To examine the performance of the proposed predictor when restricting low false positive rates, the precision-recall curve was also plotted, which plots precision (the fraction of TP in all predicted positives) against recall (sensitivity). The area under the precision-recall curve (AUPRC) was also used to examine the performance of the proposed method when restricting low false positive rates.

## Additional Information

**How to cite this article**: Chen, W. *et al.* RAMPred: identifying the N^1^-methyladenosine sites in eukaryotic transcriptomes. *Sci. Rep.* **6**, 31080; doi: 10.1038/srep31080 (2016).

## Supplementary Material

Supplementary Information

## Figures and Tables

**Figure 1:**
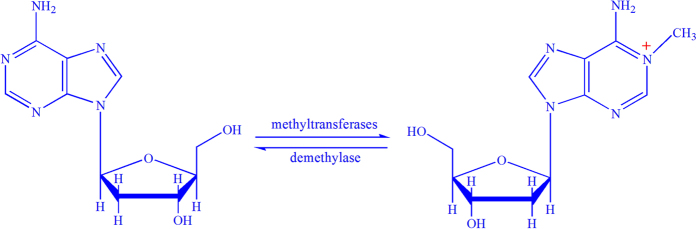
An illustration to show the N^1^-methylation and demethylation of adenosine. The formation of m_1_A is catalyzed by methyltransferases and its reversible modification is catalyzed by demethyltransferases. Besides adding a methyl group (-CH3) to the nitrogen at the 1st position of the adenosine base, N^1^-methylation also endows the modified adenosine with a positive charge as indicated in red in this figure.

**Table 1:** Predictive results of the method for identifying m^1^A sites in different species.

Species	Sn (%)	Sp (%)	Acc (%)	MCC	AUROC	AUPRC
*Human*	98.38	99.89	99.13	0.98	0.98	0.98
*Mouse*	97.46	100	98.73	0.97	0.99	0.99
*S. cerevisiae*	95.65	100	97.83	0.96	0.98	0.98
