# Supplementary material for: RAMPred: identifying the N1-methyladenosine sites in eukaryotic transcriptomes
Source: Sci Rep. 2016 Aug 11;6:31080. doi: 10.1038/srep31080 (PMC4980636; doi:10.1038/srep31080)
Supplement: Supplementary Information [file srep31080-s1.pdf]

Supplementary Materials

RAMPred: identifying the N^1-methyladenosine sites in eukaryotic transcriptomes

Wei Chen[1]*, Pengmian Feng[2], Hua Tang[3], Hui Ding[4], Hao Lin[4]*

1. Department of Physics, School of Sciences, and Center for Genomics and Computational Biology, North China University of Science and Technology, Tangshan, Tangshan 063000, China;
2. School of Public Health, North China University of Science and Technology, Tangshan, 063000, China;
3. Department of Pathophysiology, Southwest Medical University, Luzhou 646000, China.
4. Key Laboratory for Neuro-Information of Ministry of Education, Center of Bioinformatics and Center for Information in Biomedicine, School of Life Science and Technology, University of Electronic Science and Technology of China, Chengdu 610054, China;

*Corresponding authors
Wei Chen: chenweiimu@gmail.com;
Hao Lin: hlin@uestc.edu.cn;
Tel: +86-315-3725715; Fax: +86-315-3725715

Supplementary Table S1 Predictive results for identifying m[1]A based on different negative dataset for *H. sapiens*.

Dataset	*Sn* (%)	*Sp* (%)	*Acc* (%)	*MCC*
Negative set 1	97.34	99.23	98.29	0.98
Negative set 2	98.53	99.00	98.77	0.98
Negative set 3	98.21	98.96	98.59	0.97
Negative set 4	98.32	99.29	98.81	0.98
Negative set 5	98.01	99.47	98.74	0.98
Negative set 6	98.79	98.90	98.85	0.98
Negative set 8	97.89	99.77	98.83	0.98
Negative set 9	98.69	99.37	99.03	0.98

Supplementary Table S2. Predictive results for identifying m^1A based on different negative dataset for *M. musculus*.

Dataset	*Sn* (%)	*Sp* (%)	*Acc* (%)	*MCC*
Negative set 1	98.12	99.48	98.80	0.97
Negative set 2	97.68	100	98.84	0.97
Negative set 3	97.19	100	98.60	0.98
Negative set 4	96.89	98.37	97.63	0.96
Negative set 5	97.03	100	98.52	0.97
Negative set 6	97.21	99.34	98.28	0.97
Negative set 8	98.02	100	99.01	0.98
Negative set 9	97.89	100	98.95	0.98

Supplementary Table S3. Predictive results for identifying m^1A based on different negative dataset for *S. cerevisiae*.

Dataset	*Sn* (%)	*Sp* (%)	*Acc* (%)	*MCC*
Negative set 1	95.73	99.20	97.47	0.97
Negative set 2	96.29	99.18	97.74	0.97
Negative set 3	96.24	100.00	98.12	0.98
Negative set 4	95.09	98.76	96.93	0.97
Negative set 5	94.32	98.23	96.28	0.97
Negative set 6	95.88	98.79	97.34	0.97
Negative set 8	96.01	100.00	98.01	0.98
Negative set 9	95.37	99.07	97.22	0.97

Supplementary Table S4. Comparative results of different classifiers for identifying m^1A via 10-fold cross validation based on the benchmark dataset.

Method	Species	Sn (%)	Sp (%)	Acc (%)	MCC
	Human	99.27	99.47	98.78	0.98
J48	*Mouse*	96.62	98.78	97.70	0.95
	S. cerevisiae	93.79	98.14	95.96	0.92
	Human	98.49	99.89	99.09	0.98
Random Forest	*Mouse*	96.52	99.91	98.21	0.96
	S. cerevisiae	92.34	100.00	96.17	0.93
	Human	98.18	98.29	98.23	0.96
Naïve Bayes	*Mouse*	96.43	97.74	97.09	0.94
	S. cerevisiae	97.31	97.31	97.31	0.95
	Human	98.84	98.78	98.81	0.98
BayesNet	*Mouse*	97.37	98.78	98.08	0.96
	S. cerevisiae	97.72	99.79	98.76	0.98
	Human	99.38	99.91	99.14	0.99
RAMPred	*Mouse*	97.56	100.00	99.37	0.98
	S. cerevisiae	96.27	100.00	98.14	0.96

Supplementary Table S5. Comparative results of different parameters for identifying m^1A via 10-fold cross validation based on the benchmark dataset.

Parameter	Species	Sn (%)	Sp (%)	Acc (%)	MCC
RAMPred	*Human*	99.38	99.91	99.14	0.99
	Mouse	97.56	100.00	99.37	0.98
	S. cerevisiae	96.27	100.00	98.14	0.96
PseDNC (w=0.3, λ=5)[a]	*Human*	87.80	93.03	90.41	0.81
	Mouse	85.54	89.67	87.61	0.75
	S. cerevisiae	67.35	73.76	70.56	0.41

[a] The two parameters (w and λ) of pseudo dinucleotide nucleotide composition were optimized via 10-fold cross validation and set as 0.3 and 5, respectively.
